# Physical frailty, genetic predisposition, and the risks of severe non‐alcoholic fatty liver disease and cirrhosis: a cohort study

**DOI:** 10.1002/jcsm.13506

**Published:** 2024-06-18

**Authors:** Honghao Yang, Fengrong Ou, Qing Chang, Jinguo Jiang, Yashu Liu, Chao Ji, Liangkai Chen, Yang Xia, Yuhong Zhao

**Affiliations:** ^1^ Department of Clinical Epidemiology Shengjing Hospital of China Medical University Shenyang China; ^2^ Liaoning Key Laboratory of Precision Medical Research on Major Chronic Disease Shenyang China; ^3^ School of Public Health China Medical University Shenyang China; ^4^ Department of Nutrition and Food Hygiene, Hubei Key Laboratory of Food Nutrition and Safety, School of Public Health, Tongji Medical College Huazhong University of Science and Technology Wuhan China

**Keywords:** Cirrhosis, Fatty liver, Frailty, Genetic predisposition, NAFLD

## Abstract

**Background:**

Frailty, defined as a phenotype of decreased physiological reserves and diminished ability to respond to stressors, has been linked to the development of chronic diseases. Epidemiological evidence connecting frailty to non‐alcoholic fatty liver disease (NAFLD) and cirrhosis risks remain sparse. We aimed to assess the longitudinal associations of frailty with the risks of severe NAFLD and cirrhosis in middle‐aged to older adults and further explore the modification role of genetic risk on these associations.

**Methods:**

This study included a total of 398 386 participants from the UK Biobank. Incident cases of severe NAFLD and cirrhosis were ascertained through linked hospital records and death registries. Frailty status was assessed by a modified version of the frailty phenotype, encompassing five key components: weight loss, tiredness, physical activity, gait speed, and grip strength. Participants were classified as pre‐frailty if they met one or two of these criteria, and as frailty if they met three or more. Genetic predisposition to NAFLD and cirrhosis was estimated by genetic risk score (GRS) and further categorized into high, intermediate, and low genetic risk levels according to tertiles of GRSs. Cox proportional hazards regression model was employed to estimate the hazard ratios (HRs) and 95% confidence intervals (CIs) for their associations.

**Results:**

The mean (standard deviation) age of the study population was 56.6 (8.03) years. 214 408 (53.8%) of the participants was female; 14 924 (3.75%) of participants met the criteria for frailty, 170 498 (42.8%) for pre‐frailty, and 212 964 (53.5%) for non‐frailty. Over a median follow‐up of 12.0 years, we documented 4439 incident severe NAFLD and 3323 incident cirrhosis cases, respectively. Compared with non‐frailty, both pre‐frailty (HR: 1.50; 95% CI: 1.40–1.60) and frailty (HR: 1.98; 95% CI: 1.77–2.21) were associated with increased risk of NAFLD. Similar associations were observed for cirrhosis, the corresponding HRs (95% CIs) for non‐frailty, pre‐frailty, and frailty were 1.00 (reference), 1.29 (1.20, 1.38), and 1.90 (1.66, 2.18). Such associations were consistent across all genetic risk levels, with no observed interactions between frailty and GRSs (all *P* for interactions ≥0.10). Compared with participants with frailty and a low level of genetic risk, the greatest risk increasement in developing severe NAFLD (HR: 3.36; 95% CI: 2.83–3.99) and cirrhosis (HR: 2.81; 95% CI: 2.29–3.44) was both observed in those with frailty and a high level of genetic risk.

**Conclusions:**

Our findings indicate that frailty is a significant predictor of severe NAFLD and cirrhosis, irrespective of genetic predisposition.

## Introduction

Non‐alcoholic fatty liver disease (NAFLD) is now the predominant cause of chronic liver disease worldwide.^1^ It can progress to non‐alcoholic steatohepatitis (NASH) and, in severe instances, lead to cirrhosis.^1^ The global surge in the ageing population, coupled with a rising prevalence of comorbidities like diabetes, hyperlipidemia, obesity, and hypertension, has amplified the incidence of NAFLD.^2^, ^3^ However, no treatments for NAFLD have gained approval. Therefore, there is a pressing need to identify the modifiable risk factors for preventing the onset of NAFLD and its further progression to liver cirrhosis.

Frailty represents the most challenging manifestation of population ageing.^4^ It is a state of increased vulnerability to poor resolution of homoeostasis after a stressor event,^4^ which may increase risk of adverse outcomes, such as falls,^5^ disability,^6^ and even death.^6^, ^7^ Accumulated evidence suggests a potential nexus between frailty and NAFLD, given their shared common risk factors and pathological mediators such as ageing, chronic inflammation,^8^, ^9^ and impaired intestinal permeability and gut microbiota.^2^, ^10^ Prior research has shown that frailty is associated with increased incidence of progression, hospitalization, transplant delisting, and mortality in patients with cirrhosis.^11^, ^12^ However, epidemiological evidence on the longitudinal associations between frailty and risks of NAFLD and cirrhosis is still limited. Moreover, while both environmental and genetic elements influence the onset of NAFLD and cirrhosis,^13^ it remains unknown that whether the genetic predisposition may modify these associations.

In light of these gaps, in this study, we aimed to evaluate the longitudinal associations between frailty and risks of severe NAFLD and cirrhosis among middle‐aged to older adults from the UK Biobank study. In addition, we further investigated the interactive effect between genetic predisposition and frailty, as well as the effect of their joint exposure, on the risks of severe NAFLD and cirrhosis.

## Method

### Study population

Participants in the present study were sourced from the UK Biobank, which is a large and well‐designed prospective cohort study including more than 500 000 participants, aged 37 to 73 years, from 22 assessment centres across England, Scotland, and Wales between 2006 and 2010. Participants provided health‐related data through a touchscreen questionnaire survey, physical measurements, face‐to‐face interview, medical diagnosis, and various samples (e.g., blood urine and saliva). The North West Multicentre Research Ethical Committee Study approved the UKB study (11/NW/0382), and all participants provided written informed consent.

Data from 502 489 participants were available for our study. We excluded participants who were younger than 40 years (*n* = 110), not available to genetic information (*n* = 15 205) or had mismatch between genetic sex and self‐reported gender (*n* = 367), or not of European descent (*n* = 28 038) because the genetic instrument we used was constructed in White participants. Those who had incomplete data on frailty assessment (*n* = 44 002) or other covariates (*n* = 7781) were also excluded. We further excluded participants with prevalent severe NAFLD, cirrhosis, other liver disease (e.g., liver failure, hepatocellular carcinoma, liver transplant status, alcoholic liver disease, and viral hepatitis), or alcohol/drug abuse (*n* = 7608) at/before baseline (details of these diseases are provided in the *Table* *S1*). In addition, 992 participants were excluded due to loss to follow‐up. Finally, a total of 398 386 participant was included in the final analyses (flowchart of participant selection, Figure S1).

### Assessment of frailty

Consistent with prior studies,^14^, ^15^ frailty was assessed by an adaptation of Fried phenotype,^16^ which encompasses five criteria: weight loss, low grip strength, slow walking pace, low physical activity (PA), and exhaustion. Grip strength was assessed using a Jamar J00105 hydraulic hand dynamometer. Both right‐ and left‐hand grip strength were measured, and the higher of two values was selected. Low grip strength was based on cutoffs adjusted for sex and body mass index (BMI) (detailed cutoffs are provided in *Table* *S2*). The remaining four criteria were collected via a touchscreen questionnaire. Detailed methods for assessment of these four components have been presented previously or can be also seen in *Table* *S2*.^14^, ^15^ Based on participants' responses, we defined (1) ‘Yes, lost weight’ as weight loss; (2) ‘Slow pace’ as slow walking pace; (3) ‘More than half the days’ or ‘Nearly every day’ of feeling tired or having little energy as exhaustion. For individual component, participants responded other options were considered not having the corresponding frailty phenotype. For assessment of PA, participants who reported either no activity or only light activity with a frequency of once per week or less were deemed to have low PA.

In line with previous research,^14^, ^15^ based on the number of frailty phenotype criteria they met, participants were classified into non‐frail (0), pre‐frail (1 or 2), and frail (3 or more) status.

### Assessment of outcome

Severe NAFLD was defined as hospitalization or death due to severe NAFLD or NASH and were obtained through linked hospital admissions and death certificates databases.^17^, ^18^ Outcomes were classified using the Tenth International Classification of Diseases (ICD‐10) codes. Using ICD‐10, and the latest Expert Panel Consensus Statement,^19^ severe NAFLD (including NASH) was defined as ICD‐10 K76.0 and K75.8 (*Table* *S1*).^18^ Cirrhosis was defined as ICD‐10 K74.1, K74.2, K74.6, I85.9, I98.2, I86.4, I85.0, I98.3, R18, K76.6, and K76.7 (*Table* *S1*).^18^

### Assessment of covariates

Demographic information (e.g., sex, age, and ethnicity), socioeconomic data (e.g., household income, education, and Townsend deprivation index), and lifestyles (e.g., smoking status and alcohol consumption) were assessed by a touchscreen questionnaire at baseline. Anthropometric indexes (height, weight, and waist circumference [WC]) were measured by trained and experienced nurses. Body mass index (BMI) was calculated as weight (kg)/height squared (m^2^). Hypertension was defined as a systolic blood pressure ≥140 mmHg and/or a diastolic blood pressure ≥90 mmHg, self‐reported hypertension, or antihypertensive medication use. Diabetes was defined as fasting glucose ≥7.0 mmol/L, glycated haemoglobin (HbA_1c_) ≥ 48.0 mmol/L, self‐reported diabetes, or a history of diabetes medication use. Hyperlipidaemia was defined as total cholesterol ≥5.17 mmol/L, triglycerides ≥1.70 mmol/L, low‐density lipoprotein cholesterol ≥3.37 mmol/L, self‐reported hyperlipidemia, or a history of medication use for hyperlipidaemia. To evaluate the overall dietary quality, we developed a healthy diet score by combining five common elements of healthy dietary patterns, including vegetables, fruits, fish, unprocessed meat, and processed meat.^20^ We defined favourable diet factors as (1) vegetables intake ≥median; (2) fruits intake ≥median; (3) fish intake ≥median; (4) unprocessed meat ≤median; (5) processed meat ≤median. Each diet factor was assigned one point if it met the definitions of the favourable diet factors and zero point if not. Total healthy diet scores ranged from 0 to 5 and higher scores indicated healthier diets.

### Calculation of the genetic risk score for severe non‐alcoholic fatty liver disease and cirrhosis

We selected five single nucleotide polymorphisms (SNPs) genome‐wide significantly associated with NAFLD (rs738409, rs58542926, rs641738, rs1260326, and rs72613567) and six SNPs associated with liver cirrhosis (rs2642438, rs72613567, rs58542926, rs738409, rs1800562, and rs28929474) in European descent participants.^21^, ^22^, ^23^ Based on the selected SNPs, the genetic risk score (GRS) for NAFLD and cirrhosis was calculated separately as follows: GRS = (β1 × SNP1 + β2 × SNP2 + … + βn × SNPn)*(N/sum of the β coefficients), where SNPn is the risk allele number of each SNP. A higher GRS score indicated a higher genetic predisposition to NAFLD or cirrhosis. For further analysis, we divided participants into three groups (low, intermediate, and high risk for severe NAFLD or cirrhosis) according to tertiles of severe NAFLD‐GRS or cirrhosis‐GRS.

### Statistical analyses

In the current study, we employed a complete case approach. The baseline characteristics of participants were shown as mean (standard deviation [SD]) for continuous variables and percentages for categorical variables according to frailty status (non‐frail, pre‐frail, and frail). Person‐years were calculated from the date of the attendance to the date of the first diagnosis of each outcome (severe NAFLD and cirrhosis), death, drop out, or the end endpoint of follow‐up (30 September 2021 for centres in England; 28 February 2018 for centres in Wales; and 31 July 2021 for centres in Scotland). Cox proportional hazards regression model was employed to evaluate the associations between the frailty and the risks of severe NAFLD and cirrhosis with the non‐frail as the reference. We adopted the Schoenfeld residual method to check the proportional hazard assumption and no violation was found. Three models were developed. Model 1 was adjusted for age, sex, and BMI. Model 2 was further adjusted for smoking status, alcohol consumption, education level, Townsend deprivation index, and a healthy diet score. Model 3 was additionally adjusted for hypertension, diabetes, hyperlipidaemia, cancer, cardiovascular disease (CVD), GRS, the first 10 principal components of ancestry, and the genotype measurement batch. Tests for trend were performed by using the number of frailty phenotypes as a continuous variable in the Cox models. In addition, we examined the dose–response associations between the number of frailty phenotypes and the risks of severe NAFLD and cirrhosis using the restricted cubic spline analysis. For individual components of frailty analyses, all of the five frailty phenotypes were included in Cox models simultaneously.

The interaction between frailty and genetic risk was tested by adding a multiplicative interaction term into the fully adjusted model. We also estimated the associations of frailty and incident of severe NAFLD and cirrhosis stratified by the categories of genetic risk. The joint association between the frailty status and genetic risk with severe NAFLD and cirrhosis was assessed by defining a combined variable with 3 × 3 groups among participants with any frailty status, where the lowest risk combination, the low genetic risk and non‐frailty, was the reference group.

We conducted several secondary analyses to test the robustness of our results. First, to assess whether the associations of frailty status with the risks of severe NAFLD and cirrhosis differed by population subgroup, we stratified the participants by potential effect modifiers including sex, age (<60 or ≥60 years), and alcohol intake (never/special occasions or regular drinking). Second, we excluded participants who were diagnosed with cancer or CVD at baseline. Third, to lessen the effect of reverse causality, we excluded participants who developed severe NAFLD or cirrhosis within the first 2 or 5 years of follow‐up. Fourth, to minimize the potential impact of early‐stage liver function impairment on our results, we further adjusted for baseline serum levels of alanine aminotransferase (ALT) and aspartate aminotransferase (AST), which are sensitive indicators of early hepatocellular damage.^24^ Fifth, given that NAFLD is a leading cause of liver‐related mortality and hepatocellular carcinoma,^25^ we captured a wide range of NAFLD‐related outcomes by defining new‐onset severe liver diseases, including liver failure, hepatocellular carcinoma, and deaths related to these conditions (codes can be seen in *Table* *S1*), as a secondary outcome.^18^

All statistical analyses were conducted using SAS version 9.4 (SAS Institute Inc) and R version 4.0.2. A two‐sided *P*‐value <0.05 was considered statistically significant.

## Results

### Baseline characteristics of participants

A total of 398 386 participants (mean [SD] age: 56.6 [8.03] years; 214 408 females [53.8%]) were enrolled. Overall, 14 924 (3.75%) met the criteria for frailty, 170 498 (42.8%) for pre‐frailty, and 212 964 (53.5%) for non‐frailty. During a median of 12.0 years of follow‐up, we documented 4439 incident severe NAFLD cases (cases per 10 000 person‐years: 9.47) and 3323 incident cirrhosis cases (cases per 10 000 person‐years: 7.08), respectively. Table 1 displays the baseline characteristics of participants according to frailty phenotypes. Participants who were frail tended to be women, were a little older and higher in education level, appeared to be more materially deprived, had a higher proportion of current smokers but lower alcohol consumption, ate a less healthy diet, were higher in BMI, SBP, FBG, HbA_1c_, TC, TG, and LDL‐C, and had higher prevalence of diabetes, hypertension, hyperlipidaemia, CVD, and cancer.

**Table 1:** Baseline characteristics of participants according to frailty status (*n* = 398 386)

Characteristics	Frailty status			*P* value^a^
	Non‐frail (*n* = 212 964)	Pre‐frail (*n* = 170 498)	Frail (*n* = 14 924)	
Socio‐demographics				
Sex (female), *n* (%)	109 911 (51.6)	95 211 (55.8)	9286 (62.2)	<0.0001
Age (years)	55.9 (8.02)^b^	57.3 (7.99)	58.4 (7.47)	<0.0001
Education level, *n* (%)				<0.0001
High	26 961 (12.7)	17 180 (10.1)	860 (5.76)	
Intermediate	104 802 (49.2)	76 614 (44.9)	5061 (33.9)	
Low	81 201 (38.1)	76 704 (45.0)	9003 (60.3)	
Townsend deprivation index	−1.83 (2.77)	−1.30 (3.04)	0.10 (3.47)	<0.0001
Lifestyles				
Smoking status, *n* (%)				<0.0001
Current smoker	18 265 (8.58)	18 510 (10.9)	2858 (19.2)	
Ex‐smoker	74 378 (34.9)	61 942 (36.3)	5626 (37.7)	
Non‐smoker	120 321 (56.5)	90 046 (52.8)	6440 (43.1)	
Alcohol consumption, *n* (%)				<0.0001
Never	9522 (4.47)	12 616 (7.40)	2601 (17.4)	
Special occasions only	17 100 (8.03)	20 903 (12.3)	3421 (22.9)	
≤2 times/week	78 249 (36.7)	65 981 (38.7)	5316 (35.6)	
≥3 times/week	108 093 (50.8)	70 998 (41.6)	3586 (24.0)	
Healthy diet score	3.19 (1.18)	3.13 (1.21)	2.85 (1.23)	<0.0001
Anthropometric indexes				
BMI (kg/m^2^)	26.7 (4.20)	27.8 (4.99)	30.7 (6.77)	<0.0001
Blood pressure and biochemistry				
SBP (mmHg)	139.9 (19.6)	139.6 (19.6)	139.4 (20.0)	<0.0001
DBP (mmHg)	83.1 (10.7)	82.7 (11.0)	82.0 (11.5)	<0.0001
FBG (mmol/L)	5.03 (0.97)	5.16 (1.33)	5.51 (2.01)	<0.0001
HbA1c (mmol/L)	35.2 (5.29)	36.4 (7.00)	39.3 (10.5)	<0.0001
TC (mmol/L)	5.77 (1.10)	5.66 (1.16)	5.43 (1.27)	<0.0001
TG (mmol/L)	1.70 (1.00)	1.78 (1.03)	2.02 (1.16)	<0.0001
LDL‐C (mmol/L)	3.61 (0.85)	3.54 (0.88)	3.39 (0.95)	<0.0001
Individual history of disease, *n* (%)				
Diabetes	8304 (4.03)	12 886 (8.13)	2819 (19.9)	<0.0001
Hypertension	110 521 (53.7)	89 979 (56.8)	9328 (65.8)	<0.0001
Hyperlipidaemia	162 466 (78.9)	124 950 (78.8)	11 340 (79.9)	<0.0001

BMI, body mass index; DBP, diastolic blood pressure; FBG fasting blood glucose; HbA1c, glycated haemoglobin; LDL‐C, low‐density lipoprotein cholesterol; SBP, systolic blood pressure; TC, total cholesterol; TG, triglycerides.

^a^ Analysis of covariance or Chi‐square test.

^b^ Continuous variables were expressed as mean (standard deviation) and categorical variables were expressed as number (percentages).

### Independent associations of frailty and genetic risk score with the risks of severe non‐alcoholic fatty liver disease and cirrhosis

Table 2 shows the associations between frailty and the risks of severe NAFLD and cirrhosis. In the age‐, sex‐, and BMI‐adjusted model, both frailty (hazard ratio [HR] and 95% confidence interval [CI]: 2.63 [2.37, 2.93] for severe NAFLD; 2.38 [2.08, 2.71] for cirrhosis) and pre‐frailty (HRs [95% CIs]: 1.64 [1.54, 1.75] for severe NAFLD; 1.37 [1.27, 1.47] for cirrhosis) were associated with higher risks of severe NAFLD and cirrhosis compared with the non‐frailty. After further adjusting for lifestyles, education level, and Townsend deprivation index in model 2 or further adjustments for comorbidities and genetic predisposition in model 3, the observed associations were attenuated but reminded significant and the multivariate‐adjusted HRs and CIs of incident severe NAFLD and cirrhosis were 1.00 (reference) for non‐frailty, 1.50 (1.40, 1.60) and 1.29 (1.20, 1.38) for pre‐frailty, and 1.98 (1.77, 2.21) and 1.90 (1.66, 2.18) for frailty, respectively. The restricted cubic splines showed a non‐linear association between frailty and risk of severe NAFLD (*P* value for non‐linear <0.001), but linear association of frailty with risk of cirrhosis (*P* value for non‐linear = 0.46) (*Figure* *S2*). The results of the component analysis showed that each of five components were independently with risks of severe NAFLD and cirrhosis (*Table* *3*). Compared with their counterparts, the HRs (95% CI) of incident severe NAFLD and cirrhosis were 1.12 (1.03, 1.22) and 1.37 (1.23, 1.52) for low physical activity; 1.33 (1.25, 1.42) and 1.14 (1.05, 1.23) for low grip strength; 1.34 (1.24, 1.44) and 1.18 (1.07, 1.31) for exhaustion; 1.25 (1.14, 1.36) and 1.35 (1.21, 1.50) for slow gait speed; and 1.22 (1.13, 1.31) and 1.22 (1.11, 1.33) for weight loss.

**Table 2:** Associations between frailty status and the risks of severe NAFLD and cirrhosis (*n* = 398 386)

	Frailty status			*P* for trend^a^
	Non‐frail (*n* = 212 964)	Pre‐frail (*n* = 170 498)	Frail (*n* = 14 924)	
NAFLD				
Cases, *n*	1538	2393	508	‐
Person‐years	2 519 191	1 999 874	168 825	‐
Model 1^c^	1.00 (reference)	1.64 (1.54, 1.75)^b^	2.63 (2.37, 2.93)	<0.0001
Model 2^d^	1.00 (reference)	1.54 (1.44, 1.64)	2.16 (1.94, 2.41)	<0.0001
Model 3^e^	1.00 (reference)	1.50 (1.40, 1.60)	1.98 (1.77, 2.21)	<0.0001
Cirrhosis				
Cases, n	1379	1658	286	‐
Person‐years	2 521 689	2 004 908	170 183	‐
Model 1^c^	1.00 (reference)	1.37 (1.27, 1.47)	2.38 (2.08, 2.71)	<0.0001
Model 2^d^	1.00 (reference)	1.32 (1.23, 1.42)	2.11 (1.84, 2.42)	<0.0001
Model 3^e^	1.00 (reference)	1.29 (1.20, 1.38)	1.90 (1.66, 2.18)	<0.0001

BMI, body mass index; CVD, cardiovascular disease; GRS, genetic risk score; NAFLD, non‐alcoholic fatty liver disease.

^a^ Calculated by using the number of frailty phenotypes as a continuous variable.

^b^ Hazard ratio (all such values).

^c^ Model 1 was adjusted for age, sex, and BMI.

^d^ Model 2: Model 1 + education level, Townsend deprivation index, smoking status, drinking status, and a healthy diet score.

^e^ Model 3: Model 2 + hypertension, diabetes, hyperlipidemia, CVD, cancer, NAFLD‐GRS (for NAFLD) or cirrhosis‐GRS (for cirrhosis), first 10 principal components of ancestry, and genotype measurement batch.

**Table 3:** Associations between individual components of frailty and the risks of severe NAFLD and cirrhosis (*n* = 398 386)

Frailty component	Model 1^a^		Model 2^b^		Model 3^c^	
	HR (95% CI)	*P* value	HR (95% CI)	*P* value	HR (95% CI)	*P* value
NAFLD						
Low physical activity	1.28 (1.18, 1.39)^d^	<0.0001	1.15 (1.06, 1.25)	0.001	1.12 (1.03, 1.22)	0.01
Low grip strength	1.39 (1.30, 1.48)	<0.0001	1.35 (1.26, 1.44)	<0.0001	1.33 (1.25, 1.42)	<0.0001
Exhaustion	1.45 (1.35, 1.57)	<0.0001	1.37 (1.27, 1.48)	<0.0001	1.34 (1.24, 1.44)	<0.0001
Slow gait speed	1.40 (1.28, 1.53)	<0.0001	1.30 (1.19, 1.41)	<0.0001	1.25 (1.14, 1.36)	<0.0001
Weight loss	1.29 (1.20, 1.39)	<0.0001	1.27 (1.18, 1.37)	<0.0001	1.22 (1.13, 1.31)	<0.0001
Cirrhosis						
Low physical activity	1.52 (1.37, 1.68)	<0.0001	1.40 (1.27, 1.56)	<0.0001	1.37 (1.23, 1.52)	<0.0001
Low grip strength	1.16 (1.08, 1.25)	0.0001	1.16 (1.07, 1.25)	0.0002	1.14 (1.05, 1.23)	0.001
Exhaustion	1.25 (1.13, 1.38)	<0.0001	1.21 (1.10, 1.34)	0.0002	1.18 (1.07, 1.31)	0.0009
Slow gait speed	1.49 (1.34, 1.66)	<0.0001	1.42 (1.27, 1.58)	<0.0001	1.35 (1.21, 1.50)	<0.0001
Weight loss	1.27 (1.17, 1.39)	<0.0001	1.28 (1.17, 1.40)	<0.0001	1.22 (1.11, 1.33)	<0.0001

Each individual component was modelled as binary variable: in or out of normal range. All of the five individual components were included in the model simultaneously.

BMI, body mass index; CVD, cardiovascular disease; GRS, genetic risk score; NAFLD, nonalcoholic fatty liver disease.

^a^ Model 1 was adjusted for age, sex, and BMI.

^b^ Model 2: Model 1 + education level, Townsend deprivation index, smoking status, drinking status, and a healthy diet score.

^c^ Model 3: Model 2 + hypertension, diabetes, hyperlipidaemia, CVD, cancer, NAFLD‐GRS (for NAFLD) or cirrhosis‐GRS (for cirrhosis), first 10 principal components of ancestry, and genotype measurement batch.

^d^ Hazard ratio (all such values).

The associations of severe NAFLD‐GRS and cirrhosis‐GRS with the risks of severe NAFLD or cirrhosis are presented in *Table* *S3*. Both higher severe NAFLD‐GRS and cirrhosis‐GRS were associated with increased risks of the corresponding outcomes and the multivariate‐adjusted HRs and 95% CIs of incident severe NAFLD and cirrhosis were 1.00 (reference) for the low genetic risk, 1.19 (1.10, 1.28) and 1.21 (1.12, 1.31) for the intermediate genetic risk, and 1.60 (1.49, 1.71) and 1.64 (1.53, 1.76) for the highest genetic risk, respectively.

### Interaction and joint effects of frailty and GRS on the risks of severe NAFLD and cirrhosis

**Figure 1:**
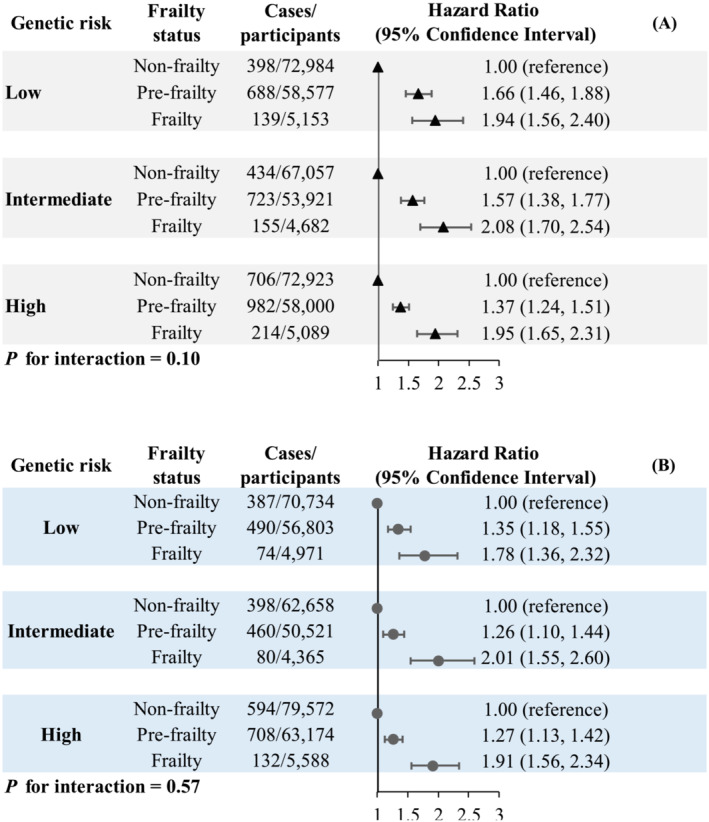
Associations between frailty status and the risks of severe NAFLD (A) and cirrhosis (B) according to genetic risk. Multivariable cox proportional regression was adjusted for age, sex, BMI, education level, Townsend deprivation index, smoking status, drinking status, a healthy diet score, hypertension, diabetes, hyperlipidaemia, CVD, cancer, NAFLD GRS (for NAFLD) or cirrhosis GRS (for cirrhosis), first 10 principal components of ancestry, and genotype measurement batch. *P* for interaction was calculated by involving the cross‐product term of frailty phenotype and NAFLD‐GRS or cirrhosis‐GRS in the Cox models. BMI, body mass index; CI, confidence interval; CVD, cardiovascular disease; GRS, genetic risk score; HR, hazard ratio; NAFLD, nonalcoholic fatty liver disease.

As shown in Figure 1, we did not observe any significant interactions between frailty and GRS on severe NAFLD or cirrhosis risk (all *P* values for interactions >0.05). The associations between frailty and risks of severe NAFLD and cirrhosis were similar across different categories of GRS.

The joint associations between frailty and GRS on the risks of incident severe NAFLD and cirrhosis are presented in Table 4. Compared with non‐frail participants with the low GRS, those with frailty and high GRS were at 236.0% and 181.0% increased risks of incident severe NAFLD (HR [95% CI]: 3.36 [2.83, 3.99]) and cirrhosis (HR [95% CI]: 2.81 [2.29, 3.44]).

**Table 4:** The association between joint effect of frailty and GRS on the risks of severe NAFLD and cirrhosis (*n* = 398 386)

Genetic susceptibility	Frailty status	Cases/participants	Hazard ratio (95% CI)^a^
NAFLD			
Low	Non‐frail	398/72 984	1.00 (reference)
	Pre‐frail	688/58 577	1.66 (1.46, 1.88)
	Frail	139/5153	1.99 (1.63, 2.43)
Intermediate	Non‐frail	434/67 057	1.20 (1.05, 1.37)
	Pre‐frail	723/53 921	1.91 (1.69, 2.16)
	Frail	155/4682	2.59 (2.14, 3.13)
High	Non‐frail	706/72 923	1.80 (1.59, 2.03)
	Pre‐frail	982/58 000	2.42 (2.15, 2.73)
	Frail	214/5089	3.36 (2.83, 3.99)
Cirrhosis			
Low	Non‐frail	387/70 734	1.00 (reference)
	Pre‐frail	490/56 803	1.34 (1.18, 1.54)
	Frail	74/4971	1.73 (1.34, 2.23)
Intermediate	Non‐frail	398/62 658	1.16 (1.01, 1.33)
	Pre‐frail	460/50 521	1.42 (1.24, 1.62)
	Frail	80/4365	2.13 (1.67, 2.72)
High	Non‐frail	594/79 572	1.37 (1.20, 1.55)
	Pre‐frail	708/63 174	1.76 (1.56, 2.00)
	Frail	132/5588	2.81 (2.29, 3.44)

BMI, body mass index; CVD, cardiovascular disease; GRS, genetic risk score; NAFLD, nonalcoholic fatty liver disease.

^a^ Multivariable Cox proportional regression was adjusted for age, sex, BMI, education level, Townsend deprivation index, smoking status, drinking status, a healthy diet score, hypertension, diabetes, hyperlipidaemia, CVD, cancer, first 10 principal components of ancestry, and genotype measurement batch.

### Secondary analyses

In stratified analyses, the associations between frailty and risks of severe NAFLD and cirrhosis were generally similar across all subgroups (*Table* *S4*). No significant interactions were observed between frailty and potential effect modifiers (all *P* values for interactions >0.05), except alcohol intake in severe NAFLD (*P* value for interaction <0.0001). The positive association between frailty and risk of severe NAFLD was more pronounced among those with regular alcohol intake.

In sensitivity analyses, further excluding participants with cancer or CVD showed similar results (*Table* *S5*). When we excluded participants who developed each outcome within the first 2 or 5 years of follow‐up, similar associations were observed (*Tables* *S6* and *S7*). The results were only slightly attenuated and remained significant after further adjusting for baseline ALT and AST (*Table* *S8*). In the analysis of frailty status with the secondary outcome risks, we found similar associations to the main analysis (*Table* *S9*).

## Discussion

In this large prospective study based on UK Biobank study, we demonstrated positive dose–response associations of physical frailty with the risks of severe NAFLD and cirrhosis in 398 386 middle‐aged to older adults. Specifically, compared with non‐frail participants, risks of severe NAFLD and cirrhosis were 98% and 90% higher among those with frailty, respectively. Moreover, these associations were present within all genetic risk categories. The highest increased risks in severe NAFLD and cirrhosis were observed in those with both frail status and high genetic risk, in contrast to non‐frail individuals with a low genetic risk.

As well‐established in previous studies, frailty is prevalent among NAFLD patients, particularly those in advanced stages of liver disease, such as cirrhosis.^11^, ^26^ It is estimated that nearly half of those with cirrhosis exhibit signs of frailty.^11^ In contrast, frailty can intensify and hasten the progression of severe NAFLD, leading to detrimental outcomes, including mortality.^11^, ^12^ However, a significant portion of the existing research is constrained by small sample sizes and cross‐sectional designs. Especially, these studies were conducted in NAFLD or cirrhotic patients. This leaves a gap in our understanding of the longitudinal associations between frailty and the risks of NAFLD and cirrhosis. Our study, therefore, offers a distinctive addition to the current body of literature. Furthermore, our results showed that both frailty and pre‐frailty were positively associated with incident severe NAFLD and cirrhosis. These findings emphasized the importance of improving frailty for the prevention of severe NAFLD and cirrhosis. For future NAFLD management guidelines, it is imperative that routine frailty assessments extend beyond just patients with severe NAFLD to also encompass the middle‐aged to older adults without NAFLD.

In the present study, we for the first time assessed whether the associations between frailty and incident severe NAFLD and cirrhosis were modified by genetic risk. We found no significant interactions between genetic risk and frailty on the outcomes. These findings suggest the strong potential benefits of routinely screening for and addressing frailty, irrespective of one's genetic predisposition. The absence of significant interactions might be attributed, in part, to the limited proportion of genetic risk explained by the included SNPs. Further studies are needed to validate our findings. Moreover, we found a stronger association between frailty and the risk of severe NAFLD among regular alcohol drinkers compared with non‐/occasional drinkers, suggesting that the impact of frailty on liver function was more dramatic in regular alcohol drinkers.

Several underlying mechanisms may explain the observed associations between frailty and the increased risks of severe NAFLD and cirrhosis. First, the loss of muscle strength and power, considered as key component of frailty,^4^ is related to decreased myokines such as irisin, which could exacerbate steatosis.^27^ Second, compelling evidence has shown that frailty is indicative of a chronic, low‐grade inflammatory state. This is evidenced by the elevated tumour necrosis factor‐a (TNF‐a), interleukin‐6 (IL‐6), and C‐reactive protein in the elderly both with frailty and pre‐frailty.^11^, ^28^, ^29^ Inflammation is a pivotal factor in the pathophysiology of NAFLD and its progression to liver cirrhosis.^8^, ^9^ Third, frailty is linked to diminished levels of growth hormone (GH) and insulin‐like growth factor‐1 (IGF‐1).^30^ GH plays a crucial role in reducing visceral fat and directly curbing hepatocellular lipogenesis, both integral in NAFLD development.^31^ IGF‐1, on the other hand, can induce cellular senescence and deactivate hepatic stellate cells, mitigating cirrhosis.^31^, ^32^ Fourth, frailty has been associated with adverse impacts on intestinal functions, such as impaired immune response, increased permeability, and reduced microbiota diversity, all of which are risk factors for NAFLD and cirrhosis.^10^, ^33^

To the best of our knowledge, the current study is the first to investigate the longitudinal associations of frailty with the risks of severe NAFLD and cirrhosis in the middle‐aged to old adults. Other strengths of this study include the cohort study design, large sample size, long‐term follow‐up, detailed information on sociodemographic factors, medical history, lifestyles, and genetic predisposition, adjustments for a wide range of confounders, and robust sensitivity analyses.

This study also has several limitations. Firstly, due to the observational nature of our study design, we cannot definitively establish a causal association between physical frailty and the risks of severe NAFLD and cirrhosis. However, the observed associations remained largely consistent even after excluding participants who developed severe NAFLD or cirrhosis within the first 2 or 5 years of follow‐up. This consistency suggests that the impact of reverse causation, if present, is likely minimal in our findings. Second, complete case analysis used in the current study may result in the exclusion of many participants with missing values for exposure assessment. Third, four of the five frailty phenotypes were self‐reported, which might lead to misclassification bias. However, it has been proved as a valid measure of frailty phenotype in the UK Biobank.^15^ Fourth, although a considerable group of confounding factors have been adjusted, we cannot rule out residual confounding by other unmeasured or unknown factors. Fifth, as this study was conducted in White mild‐to‐old adults, our findings might not be generalizable to other racial or age populations. Sixth, our study identified incident NAFLD cases using hospital inpatient records and death registration data, which might predominantly capture more advanced stages of this condition.^17^ Thus, milder cases might be missed. However, it is crucial to note that these advanced cases are of greater clinical significance,^34^ as their severity often indicates a higher likelihood of subsequent adverse outcomes.^35^ The ascertainment bias and potential milder cases will attenuate our association estimates towards the null, making our results more conservative. This suggests that our risk estimates are broadly applicable. Finally, we acknowledge the recent introduction of metabolic dysfunction‐associated steatotic liver disease (MASLD) as the proposed new terminology to replace the former NAFLD nomenclature.^36^ As the cardiometabolic criteria requested for MASLD definition were only available at baseline,^36^ we were unable to estimate incident MASLD cases during the follow‐up period. Nevertheless, given the evidence showing similar prevalence and mortality rates between NAFLD and MASLD, and that almost all patients with NAFLD align with MASLD criteria,^37^, ^38^, ^39^, ^40^ our findings of the association between frailty and the risk of severe NAFLD could be largely extrapolated under the MASLD framework.^34^ Future research, however, is needed to directly assess the association between frailty and MASLD risk to validate our findings.

## Conclusion

In conclusion, our results suggested that both frailty and pre‐frailty were associated with higher risks of severe NAFLD and cirrhosis in the middle‐aged to old adults, regardless of genetic susceptibility. Incorporating these insights into existing disease management guidelines could help mitigate the impact of severe NAFLD and cirrhosis. Furthermore, our findings provide a foundation for future research in this domain.

## Funding sources

This study was supported by the JieBangGuaShuai Project of Liaoning Province (grant number 2021JH1/1040050 to Yuhong Zhao), Young Elite Scientists Sponsorship Program by China Association for Science and Technology (grant number YESS20200151 to Yang Xia), the 345 Talent Project of Shengjing Hospital of China Medical University (grant number M0294 to Yang Xia), and the Scientific Research Project of Liaoning Province Education Department (grant number LJKMZ20221149 to Yang Xia). The funders had no role in the design and conduct of the study; collection, management, analysis, and interpretation of the data; preparation, review, or approval of the manuscript; and decision to submit the manuscript for publication.

## Conflict of interest

None of the authors has any potential conflict of interest.

## Supporting information


**Data S1.** Supporting Information

## Data Availability

UK Biobank resource data under application number 63454 was utilized for this research. Data is available in a public, open access repository. The UK Biobank data are available on application (www.ukbiobank.ac.uk/).
