# Supplementary material for: Physical frailty, genetic predisposition, and the risks of severe non‐alcoholic fatty liver disease and cirrhosis: a cohort study
Source: J Cachexia Sarcopenia Muscle. 2024 Jun 18;15(4):1491–500. doi: 10.1002/jcsm.13506 (PMC11294048; doi:10.1002/jcsm.13506)
Supplement: Supplementary file 1 — Data S1. Supporting Information [file JCSM-15-1491-s001.docx]

**Physical frailty, genetic predisposition, and the risks of severe non-alcoholic fatty liver disease and cirrhosis: A cohort study**

**Supplementary appendices**

**Supplementary references**

**Figure S1.** Flow chart for the selection of the analyzed study sample

**Figure S2.** Dose-response associations between frailty scores and the risks of severe NAFLD and cirrhosis

**Table S1.** Disease definitions used in the UK Biobank study

**Table S2.** Frailty definition and cut-off points in the UK Biobank study

**Table S3.** Associations of GRS with the risks of severe NAFLD and cirrhosis (n=398,386)

**Table S4.** Hazard ratios (95% confidence intervals) for severe NAFLD and cirrhosis according to frailty status stratified by main risk factors (n=398,386)

**Table S5.** Associations of frailty status with the risks of severe NAFLD and cirrhosis when excluding participants who had a history of cancer or CVD at baseline (n=364,200)

**Table S6.** Associations of frailty status and the risks of severe NAFLD and cirrhosis when excluding participants who developed NAFLD and cirrhosis within the first two years of follow-up

**Table S7.** Associations of frailty status and the risks of severe NAFLD and cirrhosis when excluding participants who developed NAFLD and cirrhosis within the first five years of follow-up

**Table S8.** Associations between frailty status and the risks of severe NAFLD and cirrhosis when further adjusting for baseline ALT and AST (n=398,386)

**Table S9.** Associations between frailty status and the risks of secondary outcomes (n=398,386)

**Supplementary references**

S1. Younossi ZM. Non-alcoholic fatty liver disease - A global public health perspective. *J Hepatol* 2019; **70**: 531-544.

S2. Sudlow C, Gallacher J, Allen N, Beral V, Burton P, Danesh J, et al. UK biobank: an open access resource for identifying the causes of a wide range of complex diseases of middle and old age. *PLoS Med* 2015; **12**: e1001779.

S3. Xu CQ, Mohamad Y, Kappus MR, Boyarsky B, Ganger DR, Volk ML, et al. The relationship between frailty and cirrhosis etiology: From the Functional Assessment in Liver Transplantation (FrAILT) Study. *Liver Int* 2021; **41**: 2467-2473.

S4. Van Epps P, Oswald D, Higgins PA, Hornick TR, Aung H, Banks RE, et al. Frailty has a stronger association with inflammation than age in older veterans. *Immun Ageing* 2016; **13**: 27.

S5. Tilg H, Adolph TE, Dudek M, Knolle P. Non-alcoholic fatty liver disease: the interplay between metabolism, microbes and immunity. *Nat Metab* 2021; **3**: 1596-1607.

S6. Gazzaruso C, Gola M, Karamouzis I, Giubbini R, Giustina A. Cardiovascular risk in adult patients with growth hormone (GH) deficiency and following substitution with GH--an update. *J Clin Endocrinol Metab* 2014; **99**: 18-29.

S7. Handayaningsih AE, Takahashi M, Fukuoka H, Iguchi G, Nishizawa H, Yamamoto M, et al. IGF-I enhances cellular senescence via the reactive oxygen species-p53 pathway. *Biochem Biophys Res Commun* 2012; **425**: 478-484.

S8. Miquel S, Martín R, Rossi O, Bermúdez-Humarán LG, Chatel JM, Sokol H, et al. Faecalibacterium prausnitzii and human intestinal health. *Curr Opin Microbiol* 2013; **16**: 255-261.

S9. van Tongeren SP, Slaets JP, Harmsen HJ, Welling GW. Fecal microbiota composition and frailty. *Appl Environ Microbiol* 2005; **71**: 6438-6442.

S10. Gao X, Geng T, Jiang M, Huang N, Zheng Y, Belsky DW, et al. Accelerated biological aging and risk of depression and anxiety: evidence from 424,299 UK Biobank participants. *Nat Commun* 2023; **14**: 2277.

S12. Chen L, Tao X, Zeng M, Mi Y, Xu L. Clinical and histological features under different nomenclatures of fatty liver disease: NAFLD, MAFLD, MASLD and MetALD. *J Hepatol* 2024; **80**: e64-e66.


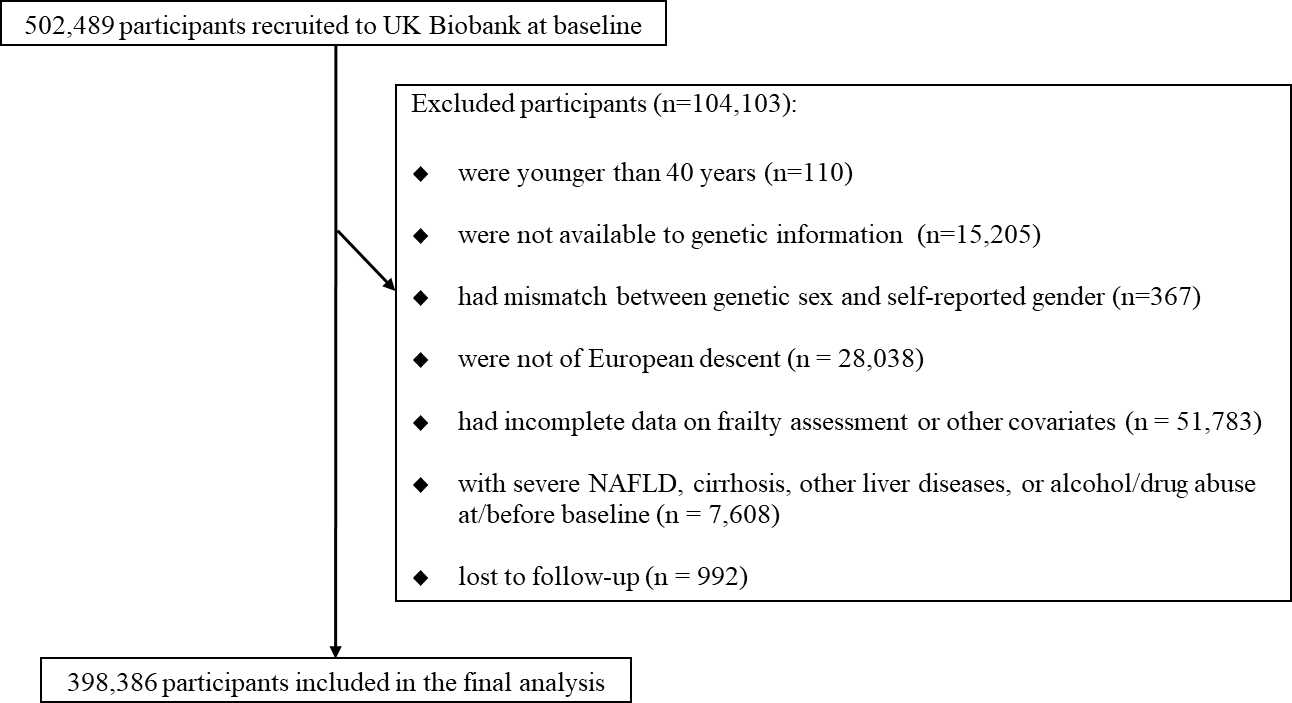


**Figure S1. Flow chart for the selection of the analyzed study sample**

**
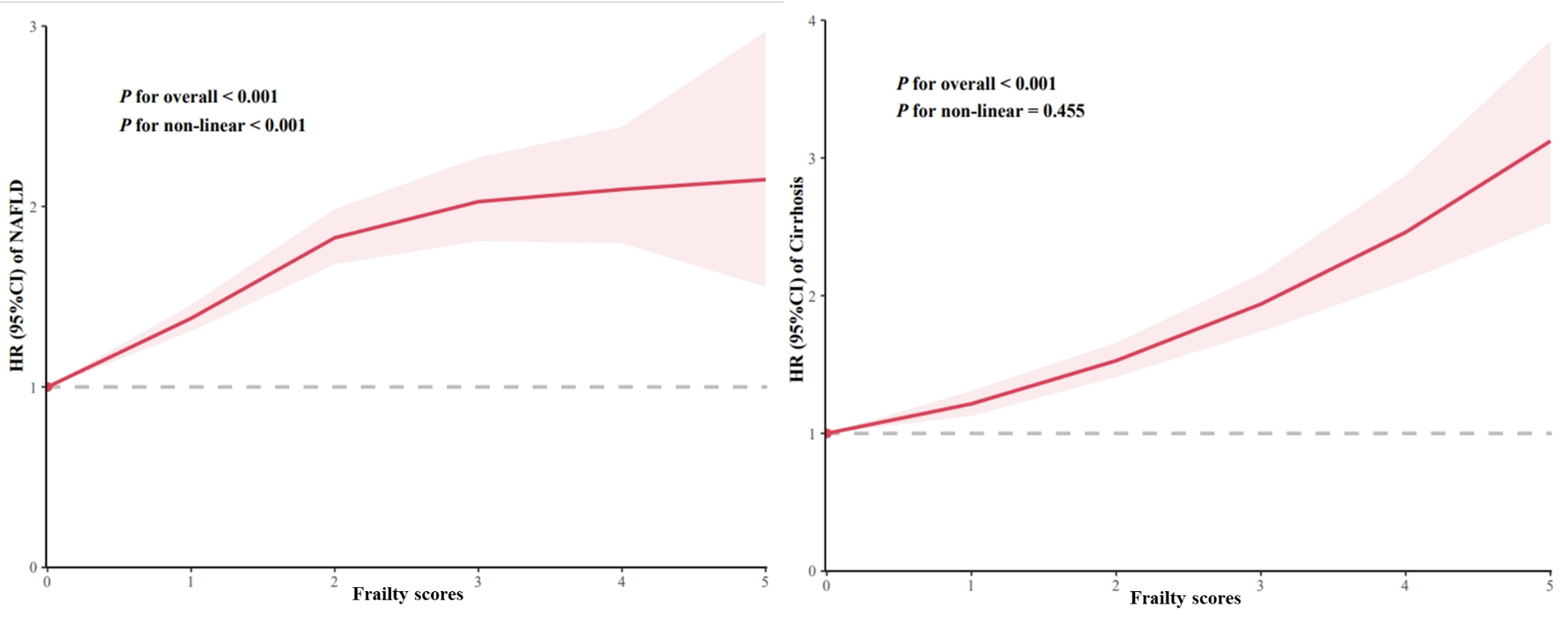
**

**Figure S2. Dose-response associations between frailty scores and the risks of severe NAFLD and cirrhosis**

Abbreviations: NAFLD, nonalcoholic fatty liver disease; BMI, body mass index; CVD, cardiovascular disease; GRS, genetic risk score. Multivariable Cox proportional regression was adjusted for age, sex, BMI, education level, Townsend deprivation index, smoking status, drinking status, a healthy diet score, hypertension, diabetes, hyperlipidemia, CVD, cancer, NAFLD GRS (for NAFLD) or cirrhosis GRS (for cirrhosis), first 10 principal components of ancestry, and genotype measurement batch.

| **Table S1**. Disease definitions used in the UK Biobank study | | | |  |
| --- | --- | --- | --- | --- |
| **Diagnosis** | **Self-report** | **ICD 10** | |  |
| **Severe NAFLD/NASH** | - | K76.0, K75.8 | |  |
| **Cirrhosis** |  |  |  |  |
| Compensated cirrhosis | - | K74.1, K74.2, K74.6, I85.9, I98.2, I86.4 | |  |
|  |  |  |  |  |
| Decompensated cirrhosis | - | I85.0, I98.3, R18, K76.7, K76.6 | |  |
|  |  |  |  |  |
| **Other liver diseases at/before baseline** | |  |  |  |
| Liver failure | - | K72.1, K72.9, K72.0 | |  |
| Hepatocellular carcinoma |  | C22.0 | |  |
| Liver transplant status | - | Z94.4 | |  |
| ALD | 1604 | K70 | |  |
| Viral Hepatitis | 1578, 1579, 1580, 1581, 1582, 1156 | B15, B16, B17, B18, B19 | |  |
|  |  |  |  |  |
| Autoimmune liver disease (AIH, PBC, PSC) | 1475, 1158, 1506 | K83.0, K74.3, K75.4 | |  |
|  |  |  |  |  |
| Hemochromatosis | 1507 | E83.1 | |  |
| Wilson | - | E.83.0 | |  |
| Alpha-1-antirypsin deficiency | 1496 | E88.0 | |  |
|  |  |  |  |  |
| Budd-Chiari | - | I82.0, K76.5 | |  |
| Chronic hepatitis, unspecified | - | K73.9, K73.2 | |  |
|  |  |  |  |  |
| Secondary or unspecified biliary cirrhosis | - | K74.4, K74.5 | |  |
|  |  |  |  |  |
| Nonspecific reactive hepatitis | - | K75.2 | |  |
|  |  |  |  |  |
| Toxic liver disease | - | K71 | |  |
| **Alcohol/drug use disorders at/before baseline** |  |  |  |  |
|  |  |  |  |  |
| Codes associated with alcohol use disorders | 1408 | F10 | |  |
|  |  |  |  |  |
| Codes associated with somatic consequence of alcohol (except ALD) | - | E24.4, G62.1, I42.6, K29.2, G31.2, G72.1, K85.2, K86.0, T51.0, T51.9, X65, Y57.3, Z50.2, Z71.4, Z72.1 | |  |
|  |  |  |  |  |
|  |  |  |  |  |
|  |  |  |  |  |
|  |  |  |  |  |
| Codes associated with drug use disorders except nicotine/caffeine | 1409 | F11-F14, F16, F18, F19 | |  |
|  |  |  |  |  |

| **Table S2**. Frailty definition and cut-off points in the UK Biobank study | | | |  |
| --- | --- | --- | --- | --- |
| Frailty components | Questions | Criteria | |  |
| **Weight loss** | Compared with one year ago, has your weight changed? | Yes, lost weight = 1 | |  |
|  |  | Others = 0 | |  |
| **Exhaustion** | Over the past two weeks, how often have you felt tired or had little energy? | “More than half the days” or “Nearly every day” = 1 | |  |
|  |  | Others = 0 | |  |
| **Physical activity** | In the last 4 weeks did you spend any time doing the following? How many times in the last 4 weeks did you do these exercises? | Physical activities include: (1) Walking for pleasure (not as a means of transport); (2) Other exercises (e.g., swimming, cycling, keep fit, bowling); (3) Strenuous sports; (4) Light DIY (e.g., pruning, watering the lawn); (5) Heavy DIY (e.g., weeding, lawn mowing, carpentry, digging).  The options of frequency include: (1) Once in the last 4 weeks; (2) 2-3 times in the last 4 weeks; (3) Once a week; (4) 2-3 times a week; (5) 4-5 times a week; (6) Every day. | |  |
|  |  |  |  |  |
|  |  |  |  |  |
|  |  |  |  |  |
|  |  | None or only light activity with a frequency of once per week or less = 1 | |  |
|  |  | Medium or heavy activity, or light activity more than once per week = 0 | |  |
| **Walking speed** | How would you describe your usual walking pace? | Slow pace = 1 | |  |
|  |  | Others = 0 | |  |
| **Grip strength** | Measured grip strength | Lowest 20% of cohort identified as low grip strength according to sex and body mass index (BMI) | |  |
|  |  |  |  |  |
|  |  | Males | Low grip strength cut-off points (kg) |  |
|  |  | BMI ≤24 kg/m^2^ | 34 |  |
|  |  | BMI 24.1 to 26 kg/m^2^ | 35 |  |
|  |  | BMI 26.1 to 28 kg/m^2^ | 36 |  |
|  |  | BMI >28 kg/m^2^ | 34 |  |
|  |  | Females | Low grip strength cut-off points (kg) |  |
|  |  | BMI ≤23 kg/m^2^ | 20 |  |
|  |  | BMI 23.1 to 26 kg/m^2^ | 20 |  |
|  |  | BMI 26.1 to 29 kg/m^2^ | 20 |  |
|  |  | BMI >29 kg/m^2^ | 20 |  |

| **Table S3**. Associations of GRS with the risks of severe NAFLD and cirrhosis (n = 398,386) ^a^ | | | | |  |
| --- | --- | --- | --- | --- | --- |
|  | Genetic risk score | | | *P* for trend ^b^ |  |
|  | Low | Intermediate | High |  |  |
| **NAFLD** |  |  |  |  |  |
| Cases, n | 1,225 | 1,312 | 1,902 | - |  |
| Person-years | 1,609,888 | 1,479,931 | 1,598,070 | - |  |
| Model 1 ^d^ | 1.00 (reference) | 1.18 (1.09, 1.28) ^c^ | 1.60 (1.49, 1.72) | <0.0001 |  |
| Model 2 ^e^ | 1.00 (reference) | 1.18 (1.09, 1.27) | 1.60 (1.49, 1.72) | <0.0001 |  |
| Model 3 ^f^ | 1.00 (reference) | 1.19 (1.10, 1.28) | 1.60 (1.49, 1.71) | <0.0001 |  |
| **Cirrhosis** |  |  |  |  |  |
| Cases, n | 1,155 | 1,223 | 2,061 | - |  |
| Person-years | 1,560,677 | 1,384,658 | 1,742,554 | - |  |
| Model 1 ^d^ | 1.00 (reference) | 1.21 (1.12, 1.31) | 1.64 (1.53, 1.77) | <0.0001 |  |
| Model 2 ^e^ | 1.00 (reference) | 1.21 (1.12, 1.31) | 1.64 (1.53, 1.77) | <0.0001 |  |
| Model 3 ^f^ | 1.00 (reference) | 1.21 (1.12, 1.31) | 1.64 (1.53, 1.76) | <0.0001 |  |
| ^a^ Abbreviations: NAFLD, nonalcoholic fatty liver disease; BMI, body mass index; CVD, cardiovascular disease; GRS, genetic risk score. | | | | |  |
| ^b^ Calculated by using the number of frailty phenotypes as a continuous variable. | | | | |  |
| ^c^ Hazard ratio (95% confidence interval) (all such values) | | | | |  |
| ^d^ Model 1 was adjusted for age, sex, and BMI. | | | | |  |
| ^e^ Model 2: Model 1+ education level, Townsend deprivation index, smoking status, drinking status, and a healthy diet score. | | | | |  |
| ^f^ Model 3: Model 2+ hypertension, diabetes, hyperlipidemia, CVD, cancer, first 10 principal components of ancestry, and genotype measurement batch. | | | | |  |
|  |  |  |  |  |  |

| **Table S4**. Hazard ratios (95% confidence intervals) for severe NAFLD and cirrhosis according to frailty status stratified by main risk factors (n = 398,386) ^a^ | | | | | |  |
| --- | --- | --- | --- | --- | --- | --- |
|  |  |  |  |  |  |  |
|  | Frailty Status | | | *P* for trend ^b^ | *P* for interaction ^c^ |  |
|  | Non-frail (n=212,964) | Pre-frail (n=170,498) | Frail (n=14,924) |  |  |  |
| **NAFLD** |  |  |  |  |  |  |
| Sex |  |  |  |  | 0.42 |  |
| Males | 1.00 (reference) | 1.44 (1.31, 1.58) ^d^ | 1.90 (1.59, 2.26) | <0.0001 |  |  |
| Females | 1.00 (reference) | 1.56 (1.42, 1.71) | 2.07 (1.79, 2.39) | <0.0001 |  |  |
| Age (years) |  |  |  |  | 0.22 |  |
| <60 | 1.00 (reference) | 1.58 (1.45, 1.73) | 2.00 (1.72, 2.33) | <0.0001 |  |  |
| ≥60 | 1.00 (reference) | 1.40 (1.26, 1.54) | 1.93 (1.65, 2.27) | <0.0001 |  |  |
| Alcohol consumption |  |  |  |  | <0.0001 |  |
| Never/special occasions | 1.00 (reference) | 1.38 (1.20, 1.58) | 1.79 (1.49, 2.14) | <0.0001 |  |  |
| Regular drinking | 1.00 (reference) | 1.52 (1.41, 1.64) | 2.14 (1.86, 2.46) | <0.0001 |  |  |
| **Cirrhosis** |  |  |  |  |  |  |
| Sex |  |  |  |  | 0.13 |  |
| Males | 1.00 (reference) | 1.29 (1.16, 1.43) | 2.06 (1.70, 2.51) | <0.0001 |  |  |
| Females | 1.00 (reference) | 1.28 (1.15, 1.42) | 1.79 (1.48, 2.17) | <0.0001 |  |  |
| Age (years) |  |  |  |  | 0.87 |  |
| <60 | 1.00 (reference) | 1.32 (1.18, 1.48) | 1.69 (1.34, 2.12) | <0.0001 |  |  |
| ≥60 | 1.00 (reference) | 1.26 (1.14, 1.39) | 2.00 (1.69, 2.38) | <0.0001 |  |  |
| Alcohol consumption |  |  |  |  | 0.64 |  |
| Never/special occasions | 1.00 (reference) | 1.31 (1.10, 1.57) | 2.01 (1.58, 2.57) | <0.0001 |  |  |
| Regular drinking | 1.00 (reference) | 1.27 (1.17, 1.37) | 1.85 (1.56, 2.19) | <0.0001 |  |  |
| ^a^ Abbreviations: NAFLD, nonalcoholic fatty liver disease; BMI, body mass index; CVD, cardiovascular disease; GRS, genetic risk score. | | | | | |  |
| ^b^ Calculated by using the number of frailty phenotypes as a continuous variable. | | | | | |  |
| ^c^ Calculated by using likelihood-ratio test. The interaction term of frailty with each potential modifier was included in the model. | | | | | |  |
| ^d^ Hazard ratios (95% confidence intervals) (all such values). Multivariable Cox proportional regression was adjusted for age, sex, BMI, education level, Townsend deprivation index, smoking status, drinking status, a healthy diet score, hypertension, diabetes, hyperlipidemia, CVD, cancer, NAFLD GRS (for NAFLD) or cirrhosis GRS (for cirrhosis), first 10 principal components of ancestry, and genotype measurement batch. | | | | | |  |
|  |  |  |  |  |  |  |
|  |  |  |  |  |  |  |
|  |  |  |  |  |  |  |

| **Table S5**. Associations of frailty status with the risks of severe NAFLD and cirrhosis when excluding participants who had a history of cancer or CVD at baseline (n = 364,200) ^a^ | | | | |  |
| --- | --- | --- | --- | --- | --- |
|  |  |  |  |  |  |
|  | Frailty Status | | | *P* for trend ^b^ |  |
|  | Non-frail | Pre-frail | Frail |  |  |
| **NAFLD** |  |  |  |  |  |
| Participants, n | 196,472 | 154,774 | 12,954 | - |  |
| Cases, n | 1,404 | 2,141 | 442 | - |  |
| Person-years | 2,325,485 | 1,820,896 | 148,117 | - |  |
| Model 1 ^d^ | 1.00 (reference) | 1.63 (1.52, 1.74) ^c^ | 2.60 (2.32, 2.92) | <0.0001 |  |
| Model 2 ^e^ | 1.00 (reference) | 1.52 (1.42, 1.63) | 2.15 (1.91, 2.41) | <0.0001 |  |
| Model 3 ^f^ | 1.00 (reference) | 1.49 (1.39, 1.60) | 1.98 (1.76, 2.23) | <0.0001 |  |
| **Cirrhosis** |  |  |  |  |  |
| Participants, n | 196,472 | 154,774 | 12,954 | - |  |
| Cases, n | 1,196 | 1,387 | 236 | - |  |
| Person-years | 2,327,825 | 1,825,513 | 149,341 | - |  |
| Model 1 ^d^ | 1.00 (reference) | 1.33 (1.23, 1.44) | 2.33 (2.01, 2.69) | <0.0001 |  |
| Model 2 ^e^ | 1.00 (reference) | 1.29 (1.19, 1.39) | 2.06 (1.78, 2.39) | <0.0001 |  |
| Model 3 ^f^ | 1.00 (reference) | 1.25 (1.16, 1.35) | 1.88 (1.62, 2.18) | <0.0001 |  |
| ^a^ Abbreviations: NAFLD, nonalcoholic fatty liver disease; BMI, body mass index; CVD, cardiovascular disease; GRS, genetic risk score. | | | | |  |
| ^b^ Calculated by using the number of frailty phenotypes as a continuous variable. | | | | |  |
| ^c^ Hazard ratio (95% confidence interval) (all such values) | | | | |  |
| ^d^ Model 1 was adjusted for age, sex, and BMI. | | | | |  |
| ^e^ Model 2: Model 1+ education level, Townsend deprivation index, smoking status, drinking status, and a healthy diet score. | | | | |  |
|  |  |  |  |  |  |
| ^f^ Model 3: Model 2+ hypertension, diabetes, hyperlipidemia, CVD, cancer, NAFLD GRS (for NAFLD) or cirrhosis GRS (for cirrhosis), first 10 principal components of ancestry, and genotype measurement batch. | | | | |  |
|  |  |  |  |  |  |

| **Table S6**. Associations of frailty status and the risks of severe NAFLD and cirrhosis when excluding participants who developed NAFLD and cirrhosis within the first two years of follow-up ^a^ | | | | |  |
| --- | --- | --- | --- | --- | --- |
|  |  |  |  |  |  |
|  | Frailty Status | | | *P* for trend ^b^ |  |
|  | Non-frail | Pre-frail | Frail |  |  |
| **NAFLD** |  |  |  |  |  |
| Participants, n | 212,899 | 170,387 | 14,891 | - |  |
| Cases, n | 1,473 | 2,282 | 475 | - |  |
| Person-years | 2,519,116 | 1,999,754 | 168,786 | - |  |
| Model 1 ^d^ | 1.00 (reference) | 1.64 (1.54, 1.76) ^c^ | 2.60 (2.33, 2.90) | <0.0001 |  |
| Model 2 ^e^ | 1.00 (reference) | 1.54 (1.44, 1.64) | 2.13 (1.90, 2.39) | <0.0001 |  |
| Model 3 ^f^ | 1.00 (reference) | 1.50 (1.40, 1.60) | 1.96 (1.75, 2.19) | <0.0001 |  |
| **Cirrhosis** |  |  |  |  |  |
| Participants, n | 212,858 | 170,345 | 14,893 | - |  |
| Cases, n | 1,273 | 1,505 | 255 | - |  |
| Person-years | 2,521,568 | 2,004,734 | 170,155 | - |  |
| Model 1 ^d^ | 1.00 (reference) | 1.34 (1.24, 1.45) | 2.28 (1.98, 2.62) | <0.0001 |  |
| Model 2 ^e^ | 1.00 (reference) | 1.30 (1.20, 1.40) | 2.02 (1.75, 2.33) | <0.0001 |  |
| Model 3 ^f^ | 1.00 (reference) | 1.26 (1.17, 1.36) | 1.83 (1.58, 2.11) | <0.0001 |  |
| ^a^ Abbreviations: NAFLD, nonalcoholic fatty liver disease; BMI, body mass index; CVD, cardiovascular disease; GRS, genetic risk score. | | | | |  |
| ^b^ Calculated by using the number of frailty phenotypes as a continuous variable. | | | | |  |
| ^c^ Hazard ratio (95% confidence interval) (all such values) | | | | |  |
| ^d^ Model 1 was adjusted for age, sex, and BMI. | | | | |  |
| ^e^ Model 2: Model 1+ education level, Townsend deprivation index, smoking status, drinking status, and a healthy diet score. | | | | |  |
|  |  |  |  |  |  |
| ^f^ Model 3: Model 2+ hypertension, diabetes, hyperlipidemia, CVD, cancer, NAFLD GRS (for NAFLD) or cirrhosis GRS (for cirrhosis), first 10 principal components of ancestry, and genotype measurement batch. | | | | |  |
|  |  |  |  |  |  |
|  |  |  |  |  |  |

| **Table S7**. Association of frailty status and the risks of severe NAFLD and cirrhosis when excluding participants who developed NAFLD and cirrhosis within the first five years of follow-up ^a^ | | | | |  |
| --- | --- | --- | --- | --- | --- |
|  |  |  |  |  |  |
|  | Frailty Status | | | *P* for trend ^b^ |  |
|  | Non-frail | Pre-frail | Frail |  |  |
| NAFLD |  |  |  |  |  |
| Participants, n | 212,744 | 170,070 | 14,804 | - |  |
| Cases, n | 1,318 | 1,965 | 388 | - |  |
| Person-years | 2,518,553 | 1,998,626 | 168,477 | - |  |
| Model 1 ^d^ | 1.00 (reference) | 1.59 (1.48, 1.70) ^c^ | 2.41 (2.13, 2.71) | <0.0001 |  |
| Model 2 ^e^ | 1.00 (reference) | 1.49 (1.39, 1.60) | 1.99 (1.76, 2.26) | <0.0001 |  |
| Model 3 ^f^ | 1.00 (reference) | 1.46 (1.36, 1.57) | 1.85 (1.64, 2.10) | <0.0001 |  |
| Cirrhosis |  |  |  |  |  |
| Participants, n | 212,594 | 169,999 | 14,838 | - |  |
| Cases, n | 1,009 | 1,159 | 200 | - |  |
| Person-years | 2,520,621 | 2,003,527 | 169,959 | - |  |
| Model 1 ^d^ | 1.00 (reference) | 1.31 (1.20, 1.42) | 2.26 (1.93, 2.64) | <0.0001 |  |
| Model 2 ^e^ | 1.00 (reference) | 1.26 (1.16, 1.38) | 1.99 (1.69, 2.34) | <0.0001 |  |
| Model 3 ^f^ | 1.00 (reference) | 1.23 (1.13, 1.34) | 1.82 (1.54, 2.14) | <0.0001 |  |
| ^a^ Abbreviations: NAFLD, nonalcoholic fatty liver disease; BMI, body mass index; CVD, cardiovascular disease; GRS, genetic risk score. | | | | |  |
| ^b^ Calculated by using continuous frailty score. | | | | |  |
| ^c^ Hazard ratio (all such value) | | | | |  |
| ^d^ Model 1 was adjusted for age, sex, and BMI. | | | | |  |
| ^e^ Model 2: Model 1+ education level, Townsend deprivation index, smoking status, drinking status, and a healthy diet score. | | | | |  |
| ^f^ Model 3: Model 2+ hypertension, diabetes, hyperlipidemia, CVD, cancer, NAFLD GRS (for NAFLD) or cirrhosis GRS (for cirrhosis), first 10 principal components of ancestry, and genotype measurement batch. | | | | |  |
|  |  |  |  |  |  |

| **Table S8**. Associations between frailty status and the risks of severe NAFLD and cirrhosis when further adjusting for baseline ALT and AST (n=398,386) ^a^ | | | | |  |
| --- | --- | --- | --- | --- | --- |
|  | Frailty Status | | | *P* for trend ^b^ |  |
|  | Non-frail (n=212,964) | Pre-frail (n=170,498) | Frail (n=14,924) |  |  |
| NAFLD |  |  |  |  |  |
| Cases, n | 1,538 | 2,393 | 508 | - |  |
| Person-years | 2,519,191 | 1,999,874 | 168,825 | - |  |
| Model 1 ^d^ | 1.00 (reference) | 1.64 (1.54, 1.75) ^c^ | 2.63 (2.37, 2.93) | <0.0001 |  |
| Model 2 ^e^ | 1.00 (reference) | 1.54 (1.44, 1.64) | 2.16 (1.94, 2.41) | <0.0001 |  |
| Model 3 ^f^ | 1.00 (reference) | 1.50 (1.40, 1.60) | 1.98 (1.77, 2.21) | <0.0001 |  |
| Model 4 ^g^ | 1.00 (reference) | 1.48 (1.38, 1.59) | 1.98 (1.77, 2.21) | <0.0001 |  |
| Cirrhosis |  |  |  |  |  |
| Cases, n | 1,379 | 1,658 | 286 | - |  |
| Person-years | 2,521,689 | 2,004,908 | 170,183 | - |  |
| Model 1 ^d^ | 1.00 (reference) | 1.37 (1.27, 1.47) | 2.38 (2.08, 2.71) | <0.0001 |  |
| Model 2 ^e^ | 1.00 (reference) | 1.32 (1.23, 1.42) | 2.11 (1.84, 2.42) | <0.0001 |  |
| Model 3 ^f^ | 1.00 (reference) | 1.29 (1.20, 1.38) | 1.90 (1.66, 2.18) | <0.0001 |  |
| Model 4 ^g^ | 1.00 (reference) | 1.27 (1.18, 1.37) | 1.88 (1.63, 2.16) | <0.0001 |  |
| ^a^ Abbreviations: NAFLD, nonalcoholic fatty liver disease; ALT, alanine aminotransferase; AST, aspartate aminotransferase; BMI, body mass index; CVD, cardiovascular disease; GRS, genetic risk score. | | | | |  |
| ^b^ Calculated by using continuous frailty score. | | | | |  |
| ^c^ Hazard ratio (all such value) | | | | |  |
| ^d^ Model 1 was adjusted for age, sex, and BMI. | | | | |  |
| ^e^ Model 2: Model 1+ education level, Townsend deprivation index, smoking status, drinking status, and a healthy diet score. | | | | |  |
| ^f^ Model 3: Model 2+ hypertension, diabetes, hyperlipidemia, CVD, cancer, NAFLD-GRS (for NAFLD) or cirrhosis-GRS (for cirrhosis), first 10 principal components of ancestry, and genotype measurement batch. | | | | |  |
|  |  |  |  |  |  |
| ^g^ Model 4: Model 3+ baseline ALT and AST. | | | | |  |

| **Table S9**. Associations between frailty status and the risks of secondary outcomes (n=398,386) ^a^ | | | | |
| --- | --- | --- | --- | --- |
|  | Frailty Status | | | *P* for trend ^b^ |
|  | Non-frail (n=212,964) | Pre-frail (n=170,498) | Frail (n=14,924) |  |
| Total severe chronic liver diseases |  |  |  |  |
| Cases, n | 337 | 429 | 97 | - |
| Person-years | 2,524,204 | 2,008,437 | 170,837 | - |
| Model 1 ^d^ | 1.00 (reference) | 1.45 (1.25, 1.67) ^c^ | 3.21 (2.53, 4.07) | <0.0001 |
| Model 2 ^e^ | 1.00 (reference) | 1.35 (1.17, 1.57) | 2.56 (2.01, 3.27) | <0.0001 |
| Model 3 ^f^ | 1.00 (reference) | 1.28 (1.10, 1.48) | 2.15 (1.68, 2.75) | <0.0001 |
|  |  |  |  |  |
| Liver failure |  |  |  |  |
| Cases, n | 240 | 291 | 71 | - |
| Person-years | 2,524,351 | 2,008,634 | 170,867 | - |
| Model 1 ^d^ | 1.00 (reference) | 1.42 (1.19, 1.68) | 3.55 (2.69, 4.69) | <0.0001 |
| Model 2 ^e^ | 1.00 (reference) | 1.31 (1.10, 1.56) | 2.76 (2.07, 3.68) | <0.0001 |
| Model 3 ^f^ | 1.00 (reference) | 1.26 (1.06, 1.50) | 2.41 (1.81, 3.22) | <0.0001 |
|  |  |  |  |  |
| Hepatocellular carcinoma |  |  |  |  |
| Cases, n | 107 | 156 | 29 | - |
| Person-years | 2,524,564 | 2,008,845 | 170,922 | - |
| Model 1 ^d^ | 1.00 (reference) | 1.54 (1.20, 1.97) | 2.42 (1.57, 3.73) | <0.0001 |
| Model 2 ^e^ | 1.00 (reference) | 1.47 (1.14, 1.89) | 2.09 (1.34, 3.25) | <0.0001 |
| Model 3 ^f^ | 1.00 (reference) | 1.33 (1.03, 1.71) | 1.63 (1.05, 2.54) | 0.0009 |
| ^a^ Abbreviations: BMI, body mass index; CVD, cardiovascular disease. | | | | |
| ^b^ Calculated by using continuous frailty score. | | | | |
| ^c^ Hazard ratio (all such value) | | | | |
| ^d^ Model 1 was adjusted for age, sex, and BMI. | | | | |
| ^e^ Model 2: Model 1+ education level, Townsend deprivation index, smoking status, drinking status, and a healthy diet score. | | | | |
| ^f^ Model 3: Model 2+ hypertension, diabetes, hyperlipidemia, CVD, and cancer. | | | | |
